# Beenome Soon: Honey Bees as a Model ‘Non-Model’ System for Comparative Genomics

**DOI:** 10.1002/cfg.288

**Published:** 2003-06

**Authors:** Jay D. Evans, Daniel B. Weaver

**Affiliations:** 1 USDA-ARS Bee Research Laboratory BARC-East Building 476 Beltsville MD 20705 USA; 2 B. Weaver Apiaries Inc. 16481 Lynn Grove Road Navasota TX 77868 USA


**Comparative and Functional Genomics**
*Comp Funct Genom* 2003; **4**: 351–352.
Published online in Wiley InterScience (www.interscience.wiley.com). **DOI:** 10.1002/cfg.288

**Feature**

### Beenome soon: honey bees as a model 'non-model' system for comparative genomics

Jay D. Evans[1]* and Daniel B. Weaver[2]
[1] *USDA-ARS Bee Research Laboratory, BARC-East Building 476, Beltsville, MD 20705, USA*
[2] *B. Weaver Apiaries Inc., 16481 Lynn Grove Road, Navasota, TX 77868, USA*

**Correspondence to:*
*Jay D. Evans, USDA-ARS Bee Research Laboratory, BARC-East Building 476, Beltsville, MD 20705, USA.*
*E-mail: evansj@ba.ars.usda*

Received: 24 January 2003
Revised: 20 February 2003
Accepted: 20 February 2003

While the explosion of genomic data and tools is fully apparent for model organisms, these tools are arguably changing paradigms most quickly in those species for which genetic studies are most challenging. One such species is the honey bee, *Apis mellifera*. New tools and resources for this species (e.g. [2,17]), an impending genome-sequencing project and new interdisciplinary teams will help bring the unique traits of honey bees into the world of comparative genomics. Several factors make honey bees a compelling choice for genomic studies. First, bees are outstanding experimental subjects for animal behaviour and learning, thanks to a well-known reward system [12], symbolic language [9,16] and phenomenal learning abilities [13]. Honey bees and other social insects also provide extreme examples of developmental switches, or polyphenisms — the generation of distinct phenotypes from an equivalent genetic background [5,6]. Associated with this switch are two traits that pique the interest of medical researchers — fertility and longevity. While workers are nearly sterile, queens lay hundreds of thousands of eggs each year, and live 10–20 times longer than workers. The causes and consequences of the queen–worker split, long known from the standpoint of nutrition, are ripe for genomic analyses.

Honey bees also show promise as a unique disease model. Since their domestication several thousand years ago, it has been recognized that bees are targeted by many of the same pests that affect human health, viz. viruses, protozoa, bacteria, fungi and other arthropods. Given this range of pathogens and a living environment that resembles culture media in humidity, warmth and available nutrients, honey bee colonies remain remarkably refractory to disease. Genomic studies clarifying how honey bees tolerate and resist disease offer exciting parallels both with other insects, such as *Drosophila* [4,8] and *Anopheles* [3], and with mammalian systems. Bees and other social insects provide an important twist on the study of disease, by allowing investigations of social elements in both the transmission and progression of disease (e.g. in termites, where diseases can be slowed by an emergent 'social immunity' caused by contact between nestmates [15]). Bees also have direct effects on human health, and genetic studies are beginning to unravel the bases of both foraging behaviour [1,11] and aggressive behaviour [7], showing fascinating parallels with *Drosophila* and other model organisms.

While the collective knowledge from thousands of years of bee breeding and research has given

Copyright © 2003 John Wiley & Sons, Ltd.

bees a rich genetic history (summarized by [10]), it has proved difficult to convince large numbers of geneticists to embrace bees as a viable study system. This hesitancy in part reflects several challenges involved with studying honey bees. Developmental studies are slowed by difficulties in rearing bees outside the hive, and within-hive manipulations often fail, due to a tendency of adult bees to remove altered larvae. In addition, bee cells have proved to be recalcitrant to *in vitro* culture. Finally, transgenesis, a requirement for any genetic model, has been achieved only recently [14]. Despite these challenges, genomic studies in honey bees are having a fine year indeed. Bees joined the queue to be the third insect for which a complete genome sequence will be made publicly available, thanks to efforts by the US National Institutes of Health, the Baylor College of Medicine's Human Genome Sequencing Center, the US Department of Agriculture (USDA) and a consortium from the honey bee research community and industry. Several labs have carried out new gene-expression studies to answer complex questions related to bee health, learning and development, as evidenced during a July 2002 meeting on honey bee biotechnology organized in Sapporo, Japan, by A. Mercer (University of Otago, NZ), B. Smith (Ohio State University, USA) and T. Kubo (University of Tokyo, Japan). Finally, the successful application of more recent genetic techniques (e.g. RNAi and other means to validate function; [2]) is speeding the investigation of the traits that make bees such compelling subjects.

Another key advance is a changing philosophy. As researchers and reviewers see the power of comparative genomics in their own systems, they are becoming more willing to accept inferences drawn from similarities between model organisms and non-model organisms, such as bees. These similarities, at the level of DNA sequence and order, transcription, proteins, or development, are indeed robust and much headway is being made by using inferences from *Drosophila* and other species to design experiments elucidating molecular details of behaviour and development in honey bees [1]. Increasingly, the direction of inference will be reversed, with bees and other non-model species informing genomics and genetics, thanks to their unique biological traits and available tools. This should be the ultimate goal of comparative genomics: the use of 'the right species for the job' to address questions of importance to fundamental and applied biology.

### References

1. Ben-Shahar Y, Robichon A, Sokolowski MB, Robinson GE. 2002. Influence of gene action across different time scales on behaviour. *Science* **296**: 741–744.
2. Beye M, Hartel S, Hagen A, Hasselmann M, Omholt S. 2002. Specific developmental gene silencing in the honey bee using a homeobox motif. *Insect Mol Biol* **11**: 527–532.
3. Christophides GK, Zdobnov E, Barillas-Mury C, *et al.* 2002. Immunity-related genes and gene families in *Anopheles gambiae*. *Science* **298**: 159–165.
4. De Gregorio E, Spellman PT, Rubin GM, Lemaitre B. 2001. Genome-wide analysis of the *Drosophila* immune response by using oligonucleotide microarrays. *Proc Natl Acad Sci USA* **98**: 12 590–12 595.
5. Evans JD, Wheeler DE. 2000. Expression profiles during honeybee caste determination. *Genome Biol* **2**: research0001. 1–1.6.
6. Evans JD, Wheeler DE. 2001. Gene expression and the evolution of insect polyphenisms. *Bioessays* **23**: 62–68.
7. Hunt GJ, Guzman-Novoa E, Fondrk MK, Page RE Jr. 1998. Quantitative trait loci for honey bee stinging behaviour and body size. *Genetics* **148**: 1203–1213.
8. Irving P, Troxler L, Heuer TS, *et al.* 2001. A genome-wide analysis of immune responses in *Drosophila*. *Proc Natl Acad Sci USA* **98**(26): 15 119–15 124.
9. Johnson RN, Oldroyd BP, Barron AB, Crozier RH. 2002. Genetic control of the honey bee (*Apis mellifera*) dance language: segregating dance forms in a backcrossed colony. *J Hered* **93**: 170–173.
10. Laidlaw HHJ, Page REJ. 1997. *Queen Rearing and Bee Breeding*. Wicwas: Cheshire, CT; 224 pp.
11. Page RE Jr, Fondrk MK, Hunt GJ, *et al.* 2000. Genetic dissection of honeybee (*Apis mellifera* L.) foraging behaviour. *J Hered* **91**: 474–479.
12. Pankiw T, Waddington KD, Page RE Jr. 2001. Modulation of sucrose response thresholds in honey bees (*Apis mellifera* L.): influence of genotype, feeding, and foraging experience. *J Comp Physiol A* **187**: 293–301.
13. Robinson GE. 1999. Integrative animal behaviour and sociogenomics. *Trends Ecol Evol* **14**: 202–205.
14. Robinson KO, Ferguson HJ, Cobey S, Vaessin H, Smith BH. 2000. Sperm-mediated transformation of the honey bee, *Apis mellifera*. *Insect Mol Biol* **9**: 625–634.
15. Traniello JF, Rosengaus RB, Savoie K. 2002. The development of immunity in a social insect: evidence for the group facilitation of disease resistance. *Proc Natl Acad Sci USA* **99**: 6838–6842.
16. von Frisch K. 1967. *The Dance Language and Orientation of Bees*. Harvard University Press:Cambridge, MA; 566 pp.
17. Whitfield CW, Band MR, Bonaldo MF, *et al.* 2002. Annotated expressed sequence tags and cDNA microarrays for studies of brain and behaviour in the honey bee. *Genome Res* **12**: 555–566.

Copyright © 2003 John Wiley & Sons, Ltd. 
